# The race for the classification of proximal periprosthetic femoral fractures : Vancouver vs Unified Classification System (UCS) - a systematic review

**DOI:** 10.1186/s12891-022-05240-w

**Published:** 2022-03-23

**Authors:** Clemens Schopper, Matthias Luger, Günter Hipmair, Bernhard Schauer, Tobias Gotterbarm, Antonio Klasan

**Affiliations:** Department for Orthopaedics and Traumatology, Kepler University Hospital GmbH, Johannes Kepler University Linz, Krankenhausstrasse 9, 4020 Linz and Altenberger Strasse 69, 4040 Linz, Austria

**Keywords:** Vancouver classification, UCS, Unified Classification System, Periprosthetic fractures

## Abstract

**Background:**

Periprosthetic femoral fractures (PFFs) represent a major cause for surgical revision after hip arthroplasty with detrimental consequences for patients. The Vancouver classification has been traditionally used since its introduction in 1995. The Unified Classification System (UCS) was described in 2014, to widen the spectrum by aiming for a more comprehensive approach. The UCS also aimed to replace the Vancouver classification by expanding the idea of the Vancouver classification to the whole musculoskeletal apparatus. After introduction of the UCS, the question was raised, whether the UCS found its place in the field of analysing PFFs. Therefore, this systematic review was performed to investigate, the use of the UCS compared to the established Vancouver classification.

**Methods:**

Medline was searched for reports published between 1 January 2016 and 31 November 2020, without language restriction. Included were original articles, irrespective of the level of evidence and case reports reporting on a PFF and using either the Vancouver or the UCS to classify the fractures. Excluded were reviews and systematic reviews.

**Results:**

One hundred forty-six studies were included in the analysis. UCS has not been used in a single registry study, giving a pooled cohort size of 3299 patients, compared to 59,178 patients in studies using the Vancouver classification. Since 2016, one study using UCS was published in a top journal, compared to 37 studies using the Vancouver classification (*p*=0.29). During the study period, the number of yearly publications remained stagnant (*p*=0.899).

**Conclusions:**

Despite valuable improvement and expansion of the latter UCS, to date, the Vancouver system clearly leads the field of classifying PFFs in the sense of the common use.

**Supplementary Information:**

The online version contains supplementary material available at 10.1186/s12891-022-05240-w.

## Introduction

Periprosthetic femoral fractures (PFFs) are one of the main causes for revision after hip arthroplasty, with an incidence ranging from 6.6-18% [1–4]. Furthermore, the incidence of periprosthetic femoral fractures is expected to increase by up to 4.6% per decade [1, 5–8] to a cumulative incidence of almost 5% [9]. PFFs can have detrimental consequences for the patient with a mortality rate of up to 11% within 1 year after surgical treatment [10]. They also represent a substantial economic burden [7, 11]. One of the key aspects after the diagnosis of PFF is the classification of the fracture, due to its therapeutic consequence, but also, development of further treatment options and comparison between specialized centres dealing with this issue [7].

The Vancouver classification, introduced in 1995 [12], is the first comprehensive approach, that clearly defines injury patterns and treatment options for this injury [13]. The classification encompasses the location of the fracture relative to the implant, the fixation of the implant to the bone after the fracture has occurred and it assesses the bone quality. Basically, this classification distinguishes A, B and C cases. “A” cases describe fractures in the intertrochanteric area, the prosthesis is considered stable. “A” cases can be subdivided into “Al”(lesser) and “Ag”(greater) entities depending on whether the lesser or the greater trochanter is involved. “B“cases describe diaphyseal fractures around or just below the prosthesis stem, the prosthesis is considered stable and unstable as well depending on the subtype. “B” cases can be subdivided into “B1”(stable stem), “B2”(loose stem) and “B3”(loose stem and substantial bone loss). “C” cases describe fractures distinct below the prosthesis stem, the prosthesis is considered stable [12]. It has been demonstrated to be valid and reproducible [1, 14]. Finally, it also provides treatment recommendations [13]. However, in concordance with the continuous increase of arthroplasty procedures [7], the occurrence of new fracture patterns came to evidence [3, 15]. As a consequence, the Unified Classification System (UCS) was introduced in 2014, expanding the idea of the well-articulated Vancouver classification to the whole musculoskeletal system [16]. Resting on the basic principle of the Vancouver classification, it additionally contains the description of interprosthetic fractures and it also comprises acetabular fractures. Thus additional modifiers were added to the Vancouver classification. A case “D” describes an interprosthetic fracture, a case “E” describes fractures of two bones supporting one prosthesis and a case “F” a fractured bone that is unreplaced but articulating with a prosthesis [17]. As the name suggests, the Unified classification was introduced to "unify" and therefore replace all eponymous classifications. Since the PPFFs are the most common type of periprosthetic fractures [18] and the UCS covers the same nomenclatural algorithm as the Vancouver classification, the UCS aims to be the most conclusive classification to describe PPFFs. Both classifications, the Vancouver system and the UCS as well, show comparable values of validity and reliability in their use, two important variables when it comes to the usability of a classification system [1, 13, 16, 19, 20]. Despite the overlapping characterizations of these classifications, it was expected that the UCS would find a definitive place in the algorithms of patient care [17]. The purpose of this systematic review was to answer this question performing a comparison, investigating the frequency of these 2 classifications for the description of PPFFs found in the orthopaedic literature.

## Material and methods

### Search strategy

This systematic review was conducted according to the Preferred Reporting Items for Systematic Reviews and Meta Analyses (PRISMA) guidelines [21]. Medline was searched for reports published between 1 January 2016 and 31 November 2020, without a language restriction. Although the UCS was proposed in 2014, we decided to exclude papers before 2016, to allow the centres to get familiar with the UCS. We included original articles, irrespective of the level of evidence, and case reports reporting on a PPFF and using either the Vancouver or the UCS to classify the fractures. We excluded reviews and systematic reviews. The search queries were: (periprosthetic) AND (fracture);((periprosthetic) AND (fracture)) AND (Vancouver););((periprosthetic) AND (fracture)) AND (unified). The search results were imported into Zotero (George Mason University, Fairfax, VA, U.S.) and duplicates excluded. The titles and abstracts were screened for the inclusion and exclusion criteria. Full texts of the included studies were accessed to retrieve the following information: Author, year of publication, size of the cohort, length of follow-up, study type (clinical, case report, biomechanical, validation, instructional) and the classification used. Finally, we investigated, whether the study has been published in the top 10% of its category in the year of its release according to the Journal Citation Reports (Clarivate Analytics, Philadelphia, PA, U.S.). References of retrieved articles were manually screened. The full list of all included studies is shown in Table 1.

**Table 1:** List of all included studies

Author	Year	Sample size	Top ten	Follow up (months)
Fan MQ [16]	2020	2412	no	0
Huang JF [3]	2018	402	no	216
Huang JF [20]	2016	228	no	178
Rupp M [22]	2019	75	no	96
Gunther T [23]	2020	75	no	52,9
Nagwadia H [24]	2018	43	no	16,5
Kim MB [25]	2017	19	no	16
Yeo [26]	2016	17	yes	28
Manara JR [27]	2018	28	no	26,4
Diaz-Dilernia F [28]	2019	54	yes	75
Karam J [29]	2020	172	no	96
Smitham [30]	2019	52	yes	39,6
Stevens [31]	2018	102	no	0
Gordon K [32]	2016	20	no	0
Joestl J [33]	2016	36	no	18,3
Lang NW [34]	2017	42	no	26
Thaler [35]	2019	40	yes	50
Trieb [36]	2016	34	no	43.2
Ghijselings S [37]	2018	8	no	60
Aleem IS [38]	2016	1	no	0
Bates BD [14]	2018	89	no	0
Herman A [39]	2019	379	no	68,4
Lochab JL [40]	2016	18	no	0
Li D [41]	2018	33	no	58
Sun [42]	2020	83	no	120
Wang [43]	2019	129	no	In-hospital-stay
Wang [44]	2019	34	no	102
Zhang [45]	2016	89	yes	12
Zheng [46]	2020	97	yes	24
Pavelka [47]	2017	83	no	min 36
Gromov K [48]	2017	1441	yes	23,7
Andriamananaivo T [49]	2020	50	no	3
Bonnevialle P [50]	2018	51	no	27,6
Cohen S [51]	2017	70	no	43
Ehlinger L [52]	2017	1	no	0
Gavanier B [53]	2017	45	no	20
Perrin [54]	2018	49	no	6
Bellova P [55]	2019	481	no	63
Brand S [56]	2016	2	no	0
Fink B [57]	2017	14	yes	52,2
Hoffmann MF [58]	2016	27	no	24
Hoffmann MF [59]	2016	109	no	25
Innmann M [60]	2017	163	yes	264
Klasan A [61]	2019	16	no	0
Müller M [62]	2019	8	no	34
Schreiner [63]	2020	18	no	18,50
Wähnert [64]	2020	8 pairs	yes	/
Wähnert [65]	2017	5 pairs	no	/
Zajonz [66]	2020	80	no	32 and 48
Zwingmann [67]	2016	70	no	40
Walcher [68]	2016	38	yes	/
Woo [69]	2016	1	no	26
Dozsai D [70]	2020	41	no	96
Dhason R [71]	2020	15	yes	0
Kittanakere SR [72]	2018	16	no	60
Baig MN [73]	2018	1	no	0
Cassidy JT [74]	2018	9	no	49,3
Fenelon C [75]	2019	138	yes	25
Sheridan [76]	2017	30	no	12 and 32
Angelini A [77]	2016	54	no	8,5
Bibiano L [78]	2019	7	no	50
Biggi S [79]	2018	207	no	12
Caruso G [79]	2017	73	no	41
Castelli A [80]	2018	24	no	36
Cottino U [81]	2019	3248	no	72
Giaretta S [82]	2019	64	no	23,1
Munegato D [83]	2020	25	no	29
Pavone [84]	2019	38	no	37,2
Randelli [85]	2018	19	no	73,8
Solarino [86]	2019	3	yes	178,8
Solarino [87]	2018	2	no	240
Spina [88]	2020	121	no	12
Spina [89]	2018	34	no	12
Kamo K [90]	2019	194	no	10
Kurinomaru N [91]	2019	1	no	4
Ochi [92]	2019	1	no	24
Okudera [93]	2020	51	no	/
Abarquero-Diezhandino A [94]	2020	1	no	0
Negrete-Corona [95]	2018	1	no	12
Bulatović N [96]	2017	23	no	14,5
Karabila MA [97]	2016	1	no	0
Duijnisveld BJ [98]	2020	52	no	12
van Rijn [99]	2020	1	yes	12
Legosz P [100]	2019	64	no	56,4
Lorkowski J [101]	2020	18	no	0
Kim SM [102]	2018	897	no	61,2
Kim YH [103]	2016	24	yes	44,4
Lee JM [104]	2018	37	no	25
Lee YK [105]	2017	19	yes	3,2
Min BW [106]	2020	63	no	5,9
Min BW [107]	2018	21	no	33,8
Park [108]	2018	5	no	103,2
Park [109]	2019	37	no	12
Shin [110]	2017	24	no	24
Won [111]	2020	10	no	4,4
Yoo [112]	2017	1	yes	2
Yoon [8]	2016	37	yes	44
Lizaur-Utrilla A [113]	2019	46	yes	39,6
Moreta J [114]	2018	43	no	60
Peiro [115]	2020	5	no	8,2
Valle Cruz [116]	2016	44	no	0
Chatziagorou [117]	2019	1381	yes	24
Chatziagorou G [118]	2018	1751	no	131
Chatziagorou G [119]	2019	465	no	67,2
Chatziagorou G [120]	2019	639	no	39,6
Mellner C [121]	2019	2528	no	47
Mukka S [122]	2016	979	yes	20
Baum C [123]	2019	16	no	120
Kabelitz M [124]	2018	109	no	1,5
Kraus MJ [125]	2017	1	no	43
Ladurner A [126]	2017	43	yes	40
Lenz M [127]	2016	12	no	0
Lenz M [128]	2016	12	no	0
Lenz M [129]	2020	14	no	0
Tsai [130]	2018	40	no	67,7
Yang [131]	2019	50	no	12
Sariyilmaz [132]	2016	15	no	/
Aslam-Pervez N [133]	2018	427	no	36
Chakrabarti D [134]	2019	32	no	21
El-Bakoury A [135]	2016	20	yes	44,6
Finlayson [136]	2018	189	no	108
Goudie [137]	2017	80	no	27
Johnson-Lynn Sarah [138]	2015	82	no	12
Jones AR [139]	2015	90	no	1,4
Moazen M [140]	2016	12	yes	0
Abdel MP [141]	2016	5417	yes	72
Abdel MP [4]	2016	32644	yes	96
Birch CE [142]	2017	6	no	18,6
Butler BA [143]	2019	1	no	0
Chalmers BP [144]	2018	11	yes	60
Christensen KS [145]	2019	1150	yes	3
Drew [146]	2016	188	yes	12
Gitajn IL [147]	2017	203	no	38,8
Griffiths S [148]	2019	49	yes	84
Johnson AJ [138]	2020	22	yes	0
Khan S [149]	2019	1	no	0
Lee S [150]	2019	53	yes	0
Marshall [151]	2017	/	no	/
O'Connell [152]	2018	30	no	/
Otero [153]	2020	129	yes	3,75
Parry JA [154]	2018	61	yes	54
Rodriguez [155]	2017	/	no	/
Scott [156]	2017	7	yes	21 and 21,7
Tibbo [157]	2019	/	yes	/
Waligora [158]	2017	10 pairs	yes	/

### Data analysis

Cohort sizes were pooled for each classification and descriptively compared. Changes in the yearly number of publications were compared using the Log-Rank (Mantel Cox) test. The ratio of publications in top 10% of journals was compared using the chi-square analysis. JASP 0.14.1 (University of Amsterdam, the Netherlands) was used for the statistical analysis.

## Results

**Fig. 1:**
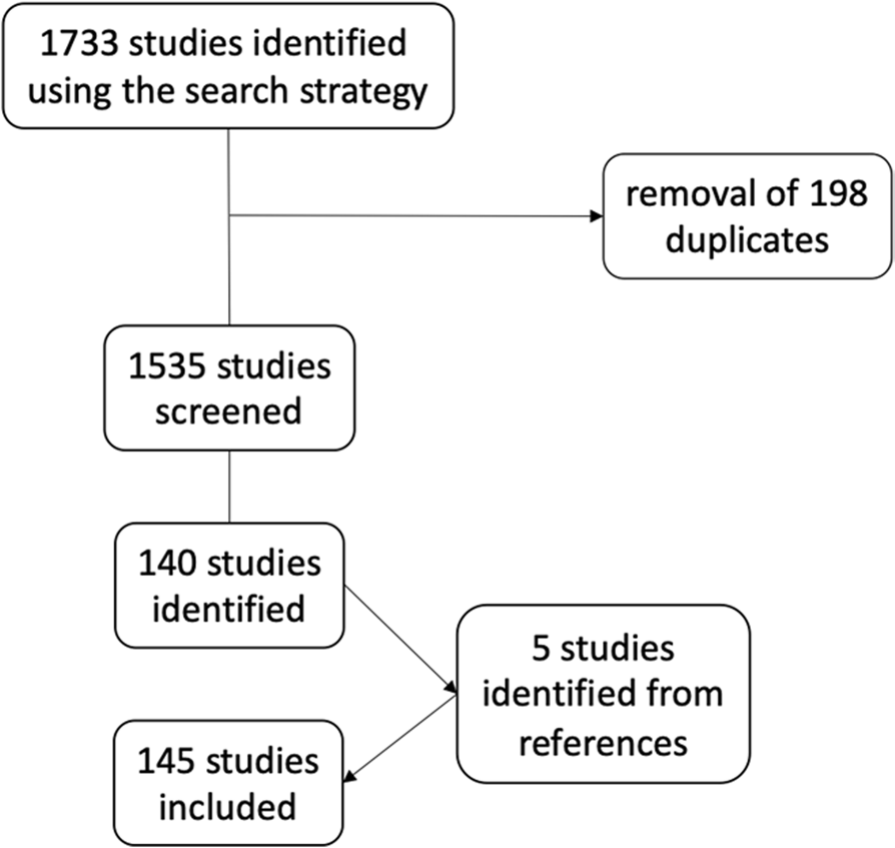
Consort diagram

**Fig. 2:**
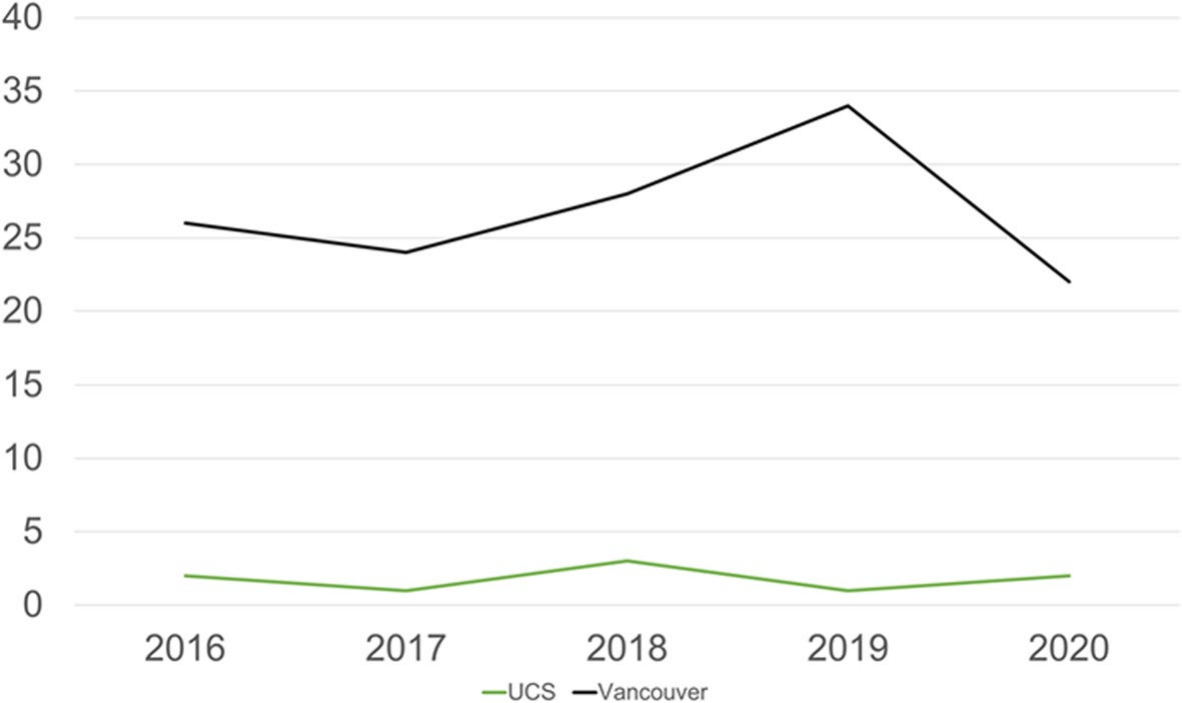
Comparison of yearly publications with Vancouver and Unified Classification between 2016 and 2020

After running the search strategy, and exclusion of duplicates, 146 studies were included for the analysis, coming from centres in 29 countries on 6 continents (Fig 1). The Unified Classification was used in 9/145 studies (6.2%). UCS has not been used in a single registry study, giving a pooled cohort size of 3299 patients, compared to 59,178 patients in the studies using the Vancouver classification. Since 2016, one study using UCS was published in a top journal, compared to 37 studies using the Vancouver classification (*p*=0.29). During the study period, the number of yearly publications remained stagnant, (*p*=0.899) (Fig 2).

## Discussion

This systematic review investigating the usage of PPFF classifications in the orthopaedic literature demonstrates that in the majority of the studies (93.8%) published since 2016 the Vancouver classification was used. Furthermore, a tendency of relevant change could not be found.

The UCS found a place in the treatment algorithms but for the most common periprosthetic fracture-the proximal femoral periprosthetic fracture-the Vancouver system remains the standard reporting classification. Although the difference is found literally in the name only and both the Vancouver and the UCS show comparable values of reliability and validity [1, 13, 16, 19, 20], it remains unclear whether the orthopaedic community is unaware of the UCS or simply “sticks” with the longer known system.

The UCS has been claimed to have had replaced the historic classifications of periprosthetic fractures [159]. This study demonstrates that this is not the case for the most common periprosthetic fracture, the PPFF. The Vancouver classification, introduced in 1995, was the first classification system to comprehensively describe periprosthetic femoral fractures including the location of the fracture with respect to the prosthesis, the bone quality of the involved bone and the information about the bony anchorage of the prosthesis [12]. The UCS aims to utilize these usable features for the whole extremity skeleton, but it still doesn’t keep up with the Vancouver classification regarding the quantitatively most important issue of the periprosthetic femoral fractures, as the latter is the most commonly used classification for the description of periprosthetic femoral fractures up to now [7].

Another reason why the UCS has been not seeing the expected usage in the literature lies to our minds in the fact that it also covers fractures of higher complexity like the description of interprosthetic fractures. Revealingly the expanded nomenclature offered by the UCS was used in only 9,6% of the clinical cases reported in our work. The incidence of the more complex PPFF cases- UCS E, D and F- is low. Since the expansion to more complex cases are the only difference to the Vancouver classification as far as the femur is concerned, this can be interpreted as an additional hindrance for the use of the UCS.

A very interesting aspect about the UCS is found in its expansion dealing with the recently added B2 type fractures involving the greater and the lesser trochanter introduced by Huang et al. These patterns were initially described by Mallory et al in 1989 [3, 15, 29, 160–162]. This expansion of the classification allows the user to more comprehensively describe the patterns involving the medial cortical wall in the case of a lesser trochanteric avulsion fracture around an implant. The stability of the medial cortical wall can be therefore classified, possibly leading to a therapeutic consequence. The modified version of the UCS also shows a higher grade of validity compared to the original classification, reaching a value of 89,8% compared to 79,7% [16]. This expansion was introduced, as the authors experienced a lack of ability to clearly distinguish between stable and unstable UCS type B fractures. The update aims to clarify the differentiation between stable and unstable cases [3], an attempt, that we doubt, as the decision still remains experience and user dependent.

The Vancouver classification on the other hand, was initially introduced for description of periprosthetic femoral fractures around a cemented stem [12]. Indeed, this classification shows high values of inter- and intraobserver reliability, but in some cases, it remains unclear, whether a cemented or cementless stem was used [13, 163, 164]. In contrast to validity values of up to 80% [13, 150, 163, 164], 25% of Vancouver type B fractures radiologically classified as stable (B1), appeared unstable intraoperatively (B2) [164]. The works of Corten et al and Lee et al also indicate that the utilization of the Vancouver classification tendential leads to misinterpretation of unstable type B cases as supposed stable findings. Both works showed a failure rate of 20% (9 out of 45 in both studies), when radiologically determined, supposed stable cases came to evidence as unstable cases intraoperatively [150, 165]. Additional works proof this tendency [166, 167]. In connection with the UCS, the ambiguity regarding the use of cemented or cementless stems becomes apparent as well [17, 19]. Some authors see potential for improvement for both classifications in this regard [150]. The authors in fact raise a doubt on the reliability of a radiologic classification used as a tool for stability assessment of a cementless, femoral stem in case of a periprosthetic femoral fracture [150]. We agree with this observation.

Furthermore, it has to be mentioned, that, although the UCS comprises an expansion of the Vancouver system, some authors still discover findings in a collective of periprosthetic femoral fractures, that are not classifiable under the use of the UCS [3]. In addition, this classification is claimed to be largely dependent on the subjective judgement of the user, especially regarding the implant stability and estimation of bone loss as well [3]. Classifying a fracture as B1 or B2 has led to a development of an algorithm, that should help with the decision of the integrity of the cement mantle and the resulting, therapeutic consequence [168].

## Conclusion

Despite valuable improvements and expansion added by the Unified Classification System to date the Vancouver classification remains the leading classification for reporting of proximal periprosthetic femoral fractures in the orthopaedic literature. Both classifications have their weaknesses due to the dependence on user experience, subjectivity or vagueness, especially when it comes to the differentiated assessment of cemented and cementless procedures.

## Supplementary Information

**Additional file 1.**

## Data Availability

The datasets used and/or analysed during the current study are available from the corresponding author on reasonable request.
